# YCharOS open antibody characterisation data: Lessons learned and progress made

**DOI:** 10.12688/f1000research.141719.1

**Published:** 2023-10-16

**Authors:** Michael S. Biddle, Harvinder S. Virk

**Affiliations:** 1 NIHR BRC-Respiratory, University of Leicester, Leicester, England, UK

**Keywords:** Antibodies, antibody validation, antibody characterisation, Open science, RRID (Research Resource Identification Initiative), YCharOS (antibody characterisation through Open Science).

## Abstract

YCharOS is a collaborative initiative aimed at characterising antibodies against the entire human proteome. As of August 2023, they have presented comprehensive knockout characterisation data for 812 antibodies and 78 proteins using techniques such as Western blot, immunoprecipitation, and immunofluorescence.

YCharOS consolidates its data into reports (one protein per report) available on Zenodo, a public repository controlled by CERN, to ensure open access. To enhance the visibility of their work, the group is progressively converting their Zenodo reports into F1000 articles, collected on the YCharOS Gateway, and indexed via PubMed. Their data is also accessible through searches on the Antibody Registry. The provided data is a valuable resource for researchers when selecting antibodies for specific applications, although certain limitations should be considered.

The data accumulated thus far has illuminated the extent of the problem when poorly performing antibodies are employed in research. While the scientific community was already aware that this was likely a widespread issue, the establishment of a collaborative open science project with industry partners introduces an innovative solution that holds the potential to yield significant returns on investment in the public interest. This potential is substantiated by the number of antibodies that have either been withdrawn or had their recommended usage altered by the vendor. However, despite the discovery of high-performing renewable antibodies for most of the studied proteins, this accounts for a tiny fraction of the human proteome and the commercial antibody market. To realise the full potential of this work, end-users must adjust their antibody procurement and usage practises in line with the provided data.

This editorial offers a guide on how individual scientists can utilise the YCharOS data, in addition to sharing the insights gained from the data thus far with the wider scientific community.

## Introduction

### Understanding and using YCharOS data

The YCharOS working group is situated at the Structural Genomics Consortium site within the Montreal Neurological Institute (The Neuro, McGill University); they perform open antibody characterisation of commercial reagent antibodies. The YCharOS characterisation pipeline has been previously described. ^1^ Here, we provide a practical guide to understanding and utilising a characterisation report. A YCharOS F1000 article commences with a specification of the cell lines used (Table 1 of every Antibody Characterization report). The process for selecting a cell line involves either acquiring it from a commercial partner or performing in-house CRISPR-Cas-9 knockout in a parental cell line (hereafter referred to as wild-type) that demonstrates sufficient expression of the protein of interest. When the gene of interest is crucial for cell survival or proliferation, knockout controls are unfeasible. In such cases, RNA knockdown controls are implemented.

After describing the cell lines used, the report then specifies the characterised antibodies (Table 2 of every Antibody Characterization report), including unique Research Resource Identifiers (RRID). Renewable antibodies, meaning they potentially have an unlimited supply with a lower risk of lot-to-lot variation, are highlighted with asterisks. These antibodies are either derived from hybridomas or are recombinant antibodies produced by plasmid transfection of cultured cell lines. Animal-derived polyclonal antibodies, on the other hand, are not renewable. When selecting an antibody, opting for a renewable option is preferred to enhance the long-term reproducibility of experiments (also refer below for the performance advantages of recombinant antibodies).

The first data figure within a YCharOS F1000 article always involves the characterisation of antibodies by Western blot. It presents a wild-type cell lysate (with sufficient expression of the protein of interest) alongside a knockout cell lysate. As for secreted proteins, centrifuged cell culture media is used instead of cell lysates. The best-performing antibodies for western blot will show bands only in the wild-type lane. A selective antibody might display multiple wild-type bands, which could represent, for example, truncated splice isoforms, multimers, post-translational modified forms of the protein-of-interest.

Antibodies with non-selective bands in the wild-type lane might still be useful if the selective signal is strong and distinguishable from the non-selective signal. On rare occasions, for specific proteins, all antibodies fail to detect the wild-type specific signal. In these cases, it is unclear if this absence represents a lack of sensitive antibodies, a problem with the knockout cell line or the protein target not being expressed within the wild type. For some genes, alternative transcription mechanisms ( *e.g.*, translation from downstream ATG sites) can still lead to the transcription and translation of a gene product, despite successful CRISPR-Cas9 edits of the target gene.

The next figure in the report shows the results of immunoprecipitation experiments for each antibody. Unlike Western blot, immunoprecipitation detects the protein in its native conformation; thus, the inability to identify the protein by Western blot does not preclude the antibody’s capacity to capture the protein of interest. The performance in immunoprecipitation is assessed by Western blot, using an antibody that has demonstrated selectivity. The immunoprecipitated fractions are presented alongside the starting material and unbound fraction. A highly efficient antibody will exhibit a robust band in the immunoprecipitated fraction, accompanied by the absence of a band in the unbound fraction. This experiment cannot be employed to infer the selectivity of an antibody by immunoprecipitation, as it does not assess whether the antibody in question also precipitates other proteins.

In the subsequent figure, an evaluation of antibody performance in immunofluorescence is presented for non-secreted proteins. The employed strategy involves a mosaic of wild-type and knockout cells, each labelled with distinct fluorescent dyes, plated together on the same coverslip or well of a 96-well plate. The outline of wild-type cells is depicted in green, with knockout cells illustrated in red. A selective antibody will exclusively stain wild-type cells, leaving knockout cells visible only by their red outline. Some reports include an automated quantification of wild type to knockout signal intensity, with a suggested threshold ratio of 1.5 to indicate reasonable selectivity. When quantification is not provided, a visual assessment can be employed.

Immunofluorescence is conducted using a standardised protocol that involves paraformaldehyde fixation along with triton X-100 as a detergent. However, if all antibodies fail to demonstrate selectivity following this protocol, an additional protocol is applied and presented. ^2^ While it is conceivable that the subpar performance of antibodies seen in this application could be protocol-specific, it is important to acknowledge that in most cases, antibodies are found with reasonable performance in immunofluorescence, indicating the protocol’s suitability for at least these antibodies. When selective antibodies are identified, it would suggest that optimising a protocol for poor-performing antibodies would be challenging. In cases where high-performing immunofluorescence antibodies are not identified, employing less frequently used immunofluorescence protocols could potentially enhance selectivity.

When using the YCharOS data to make antibody purchasing decisions, users should select the antibody with the highest selectivity (or efficiency in the case of immunoprecipitation) for their intended application, with emphasis on renewable antibodies. Notably, the YCharOS group does not assign scores to antibodies due to the dependency of their characterisation data on distinct protocols and cell lines. It is important to acknowledge that while antibodies may demonstrate impressive performance within the YCharOS pipeline, such performance does not necessarily guarantee similar performance across different experimental protocols and cell types. Therefore, we strongly advise that users consider performing genetic validation (knockdown/knockout) if feasible for their specific cell type.

### Lessons learned so far: For individual researchers

For a comprehensive analysis of the initial 614 antibodies characterised by YCharOS, we refer readers to their most recent study. ^3^ In this section, we outline our perspective on the critical insights gained. Regrettably, it must be acknowledged that the overall performance of antibodies in all applications was subpar, particularly for polyclonal antibodies. Of interest, this observation extends to immunoprecipitation experiments, contradicting the conventional assumption that polyclonal antibodies, through binding to multiple epitopes, should confer higher efficiency.

The top-performing antibodies were recombinant. However, this outcome doesn’t necessarily imply an inherent superiority of this technology. Instead, it could reflect the fact that these are newer reagents that may have undergone more rigorous quality controls before their market release. The application with the best (albeit poor) performance was western blotting, which is relatively encouraging news considering its widespread use. Notably, antibody vendors who presented genetic control data had a strong correlation with antibody performance. In the case of western blot, the inclusion of orthogonal controls (such as cell lines with varying levels of RNA) was also associated with reasonable performance, in contrast to other non-genetic controls or the absence of controls. ^3^

On the other hand, immunofluorescence performance was globally poor. ^3^ Antibodies that exhibited poor performance in immunofluorescence seldom had corroborative data in the literature, which further solidified the notion that the problem resided in the antibody’s inherent performance characteristics rather than being attributed to the staining protocol. This notion is supported by the decision of vendors, who, in numerous instances, chose to modify usage recommendations or withdraw antibodies from the market. While the presence of genetic control data on the vendor’s website showed promise as a predictor of satisfactory immunofluorescence performance, the use of orthogonal control data proved to be an unreliable predictor. ^3^

In their comprehensive analysis, YCharOS also presents the correlations between antibody performance across various applications. Despite the observed associations, it is not prudent to infer that strong performance in one application guarantees similar performance in another, for a particular antibody. Immunofluorescence performance was globally poor, and particularly the selectivity demonstrated in Western blot should not be used as evidence of selectivity in immunofluorescence or immunoprecipitation.

In conclusion, end-users can utilise the YCharOS data to select antibodies that are more likely to exhibit good performance in a particular application, if data is supportive. Additional controls are highly recommended for the specific protocols and cell types being employed. Special attention must be paid when selecting and validating an antibody for immunofluorescence, and it’s important to note that the immunoprecipitation data presented does not imply selectivity. Unfortunately, most commercial antibodies have not undergone characterisation by YCharOS. However, in some instances, alternative sources of comparable data are accessible and should be evaluated similarly.

### Lessons learned thus far: Wider scientific community

Unfortunately, the YCharOS dataset has corroborated much of the previous anecdotal evidence, some evidence from other applications, ^4^ and commentary, which has highlighted what has been referred to as the “antibody horror show”. ^5^^–^^9^ This has detrimental implications not only for individual projects and scientists ^10^^,^^11^ but also, for entire research fields, ^1^^,^^8^ resulting in a significant economic toll. ^12^ However, there are important positive aspects to be gleaned from this collaborative Open Science project. The establishment of an ecosystem where scientists and industry collaborate to rectify commercial antibody catalogues is indeed innovative. Progress can be achieved, particularly when stakeholders work together instead of trying to assign blame.

For now, YCharOS has only covered a small fraction of the human proteome. However, their data does suggest that commercial catalogues already contain a significant number of renewable antibodies necessary for studying the human proteome. ^3^ The challenge lies in scaling up their initiative and other similar initiatives to identify these antibodies. Part of the challenge stems from the research incentive landscape; their work does not directly lead to disease-related discoveries, which is where funding is primarily directed in biomedical research. It has been noted that these characterisation experiments remain too costly to be a financially viable aspect of the manufacturing process. ^3^ However, this issue does result in the drug development pipeline being inefficient and expensive. Therefore, investing in antibody characterisation could yield a substantial return on investment.

YCharOS presents an innovative model that potentially solves this problem, offering the added advantage of being a third party with no commercial interest in the outcomes of characterisation. This makes their data a less biased source compared to an antibody vendor. ^13^ However, additional resources will be needed to scale their effort, to achieve a substantial return on investment.

For their efforts to yield the desired impact, antibody end-users need to effectively utilise the provided open data. To facilitate this, YCharOS disseminates its data through platforms such as F1000, Zenodo, and the RRID portal. However, this process requires end-users to locate the data, comprehend it, and be motivated to apply it appropriately.

An opportunity exists for others to contribute similar characterisation data openly, for instance, via platforms like Biomed Resource Watch. This collective sharing of data can accelerate the progress towards attaining renewable, potent, and selective antibodies for every protein in various proteomes.

The F1000 YCharOS Gateway can be found here: https://f1000research.com/ycharos.

## Data Availability

No data is associated with this article.
